# Examining the immune signatures of SARS-CoV-2 infection in pregnancy and the impact on neurodevelopment: Protocol of the *SIGNATURE* longitudinal study

**DOI:** 10.3389/fped.2022.899445

**Published:** 2022-12-21

**Authors:** Nathalia Garrido-Torres, Lucas Cerrillos, Susana García Cerro, Alberto Pérez Gómez, Manuel Canal-Rivero, Beatriz de Felipe, Luis Alameda, Renata Marqués Rodríguez, Sergio Anillo, Julia Praena, Cristina Duque Sánchez, Cristina Roca, María Paniagua, Alvaro López Díaz, Rafael Romero-García, Peter Olbrich, Martín de Porres Puertas Albarracín, Pablo Reguera Pozuelo, Irene Luján Sosa, María Begoña Moreno Dueñas, Rocío Pineda Cachero, Lidia Zamudio Juan, Verónica García Rumi, Mercedes Guerrero Benitez, Rosario Figueroa, Antonio Manuel Martín Rendón, Antonio Partida, María Isabel Rodríguez Cocho, Carmen Gallardo Trujillo, Isabel Gallego Jiménez, Sarah García Spencer, Marta Gómez Verdugo, Cintia Bermejo Fernández, María Pérez Benito, Rafael Esteban Castillo Reina, Angela Cejudo López, Candela Sánchez Tomás, María Ángeles Chacón Gamero, Ana Rubio, Amanda Moreno Mellado, Víctor Ramos Herrero, Ella Starr, Marta González Fernández de Palacios, Elena García Victori, Antonio Pavón Delgado, Ismael Fernández Cuervo, Alejandro Arias Ruiz, Irene Esperanza Menéndez Gil, Inés Domínguez Gómez, Itziar Coca Mendoza, Rosa Ayesa-Arriola, Lourdes Fañanas, Juan C Leza, José M Cisneros, Javier Sánchez Céspedes, Ezequiel Ruiz-Mateos, Benedicto Crespo-Facorro, Miguel Ruiz-Veguilla

**Affiliations:** ^1^ Mental Health Unit, Virgen del Rocio University Hospital, Seville, Spain; ^2^ Translational Psychiatry Group, Seville Biomedical Research Institute (IBiS), Seville, Spain; ^3^ Spanish Network for Research in Mental Health CIBERSAM, ISCIII, Madrid, Spain; ^4^ Department of Psychiatry, University of Seville, Seville, Spain; ^5^ Department of Genetics, Reproduction and Maternal-Fetal Medicine, University Hospital Virgen del Rocío, Seville, Spain; ^6^ Clinical Unit of Infectious Diseases, Microbiology and Preventive Medicine, Seville Biomedical Research Institute (IBiS), Virgen del Rocío University Hospital, CSIC, University of Seville, Seville, Spain; ^7^ Congenital Immunity Disorders Group de Alteraciones Congénitas de Inmunidad, Seville Biomedical Research Institute, Seville, Spain; ^8^ Pediatrics, Infectious Diseases and Immunology Department, University Hospital Virgen del Rocío, Sevilla, Spain; ^9^ Service of General Psychiatry, Lausanne University Hospital (CHUV), Lausanne, Switzerland; ^10^ Department of Psychosis Studies, Institute of Psychiatry, Psychology & Neuroscience, King's College London, London, United Kingdom; ^11^ Department of Pediatrics, Virgen del Rocío University Hospital / Institute of Biomedicine of Seville (IBiS), Seville, Spain; ^12^ Department of Medical Physiology and Biophysics, Seville Biomedical Research Institute (IBiS), Virgen del Rocío University Hospital, CSIC, University of Seville, Seville, Spain; ^13^ Department of family medicine, Virgen del Rocío University Hospital, Primary Care Health Centers, Seville, Spain; ^14^ Department of Psychiatry, University Hospital Marqués de Valdecilla - Instituto de Investigación Marqués de Valdecilla (IDIVAL), Santander, Spain; ^15^ Department of Evolutionary Biology, Ecology and Environmental Sciences, Faculty of Biology, University of Barcelona (UB), Barcelona, Spain; ^16^ Department of Pharmacology & Toxicology, Faculty of Medicine, Universidad Complutense Madrid, CIBERSAM, Imas12, IUINQ, Madrid, Spain; ^17^ Viral Diseases and Infections in Immunodeficiencies Research Group, Institute of Biomedicine of Seville (IBiS), Virgen del Rocío University Hospital/CSIC/University of Seville, Seville, Spain

**Keywords:** pregnancy, neurodevelomental disorders, COVI-19 pandemic, SARS-CoV-2, autism (ASD), maternal mental health

## Abstract

The COVID-19 pandemic represents a valuable opportunity to carry out cohort studies that allow us to advance our knowledge on pathophysiological mechanisms of neuropsychiatric diseases. One of these opportunities is the study of the relationships between inflammation, brain development and an increased risk of suffering neuropsychiatric disorders. Based on the hypothesis that neuroinflammation during early stages of life is associated with neurodevelopmental disorders and confers a greater risk of developing neuropsychiatric disorders, we propose a cohort study of SARS-CoV-2-infected pregnant women and their newborns. The main objective of *SIGNATURE* project is to explore how the presence of prenatal SARS-CoV-2 infection and other non-infectious stressors generates an abnormal inflammatory activity in the newborn. The cohort of women during the COVID-19 pandemic will be psychological and biological monitored during their pregnancy, delivery, childbirth and postpartum. The biological information of the umbilical cord (foetus blood) and peripheral blood from the mother will be obtained after childbirth. These samples and the clinical characterisation of the cohort of mothers and newborns, are tremendously valuable at this time. This is a protocol report and no analyses have been conducted yet, being currently at, our study is in the recruitment process step. At the time of this publication, we have identified 1,060 SARS-CoV-2 infected mothers and all have already given birth. From the total of identified mothers, we have recruited 537 SARS-COV-2 infected women and all of them have completed the mental health assessment during pregnancy. We have collected biological samples from 119 mothers and babies. Additionally, we have recruited 390 non-infected pregnant women.

## Introduction

Foetus exposure to inflammation and its characteristics and severity are determining risk factors for neurodevelopmental disorders and eventually neuropsychiatric diseases [e.g., schizophrenia, autism spectrum disorders (ASD), attention deficit hyperactivity disorder] (rev. in Hagberg et al., 2015). A recent meta-analysis suggests that maternal infection during pregnancy confers an increased risk for ASD in offspring (1). Existing literature on the link between epidemics, pandemics and the increased incidence of schizophrenia spectrum disorders suggests that exposure to SARS-CoV-2 in the utero may put children born during this pandemic at risk for specific neuropsychiatric outcomes (2).

There are currently 100 million pregnant women in the world who are susceptible to being infected by SARS-CoV-2. Cohort studies of pregnant women and their descendants offer an unique opportunity to explore and anticipate the effects of SARS-CoV-2 infection on relevant neurodevelopmental aspects (3). Epidemiological studies, also carried out in pandemic periods, support the idea that the activation of the immune system during pregnancy has important consequences for foetal neurodevelopment and seems to be closely related to the subsequent developmental disorders such as schizophrenia and ASD (4, 5); There is an epidemiological association between exposure to influenza (H1N1 influenza) infection during pregnancy and an increased risk of developing neuropsychiatric disorders, suggesting that activation of the maternal immune system and an inflammatory response in the foetus (6) may be the origin of abnormal brain development (7).

The analysis of the gene expression of the offspring from mothers exposed to influenza showed the existence of an overexpression of genes associated with schizophrenia (8). However, the knowledge of the underlying pathogenic mechanisms and the associated molecular bases are not yet clear, attending that studies of this type in humans are very scarce and limited in their methodology. Several mechanisms have been proposed to explain the way in which maternal infection can interfere with brain development in offspring: (i) a systemic allostatic overload with loss of structural and functional integrity of the placenta; (ii) an activation of maternal and foetal immune responses with the production of neuronal antibodies and pro-inflammatory cytokines / chemokines and establishment of a pro-inflammatory state in the foetus and new-born; (iii) an interference in foetal neurodevelopment through the direct brain infection (6, 9).

Thanks to animal models, an even more robust immunological hypothesis has been outlined about the etiopathogenesis of various mental disorders and ASD. Some relevant neuroimmune factors found in the Central Nervous System (CNS) of murine models of maternal immunological activation (MIA) are: the activation of the microglia in a region-specific way (10), a decrease in the number of parvalbuminergic interneurons (11), alterations in the extracellular matrix (9) hyperactivity of innate immune pathways (12) changes in kynurenine metabolism (13) or alterations in cytokine levels, specifically interleukin (IL)-6, IL-10 and IL-1β, and tumour necrosis factors (TNF), specifically TNF*α* and TNF*β*. However, not all stressed or immune challenged animals, develop behavioral alterations and animal models can explain differences between vulnerable or resilient phenotypes (14).

All these alterations have been suggested as possible biological mechanisms involved in neuropsychiatric disorders (15, 16). The innate immune response of CNS cells promotes and modulates the recruitment and activation of peripheral immune cells through chemokine expression, Blood-Brain Barrier (BBB) modulation, and cell-cell interaction. In this sense, the monocyte / macrophage axis plays a fundamental role with a direct correlation between the intracellular production of pro-inflammatory cytokines in monocytes and the levels of inflammatory biomarkers (17).

It is important to highlight the role that the placenta plays in possible neurodevelopmental alterations. In a recent publication entitled, “The placenta as a window to the brain” (18), the investigators review and highlight the role of placental markers in prenatal neurodevelopmental programming. In this line of work, cohorts of new-borns with Zika virus infection are a design model when it comes to detecting alterations in the neurodevelopment of these children and their biomarkers associated with viral infection (19). Currently, there are methods to obtain stem cells from umbilical cord blood that can be later differentiated into neurons (20). Another important contribution of this proposal is the possibility of generating a biobank of umbilical cord blood samples from pregnant women with COVID-19. The samples obtained in the study proposed in this project would allow, in the future, the generation of neural progenitor lines. That would give rise to *in vitro* neuronal cultures where the cellular and molecular alterations resulting from viral infection processes, as well as stress to which mothers have been subjected during pregnancy, could be explored. In this sense, in considering a cohort of pregnancies positive for SARS-CoV-2 and a negative uninfected cohort, the longitudinal study of births in these cases will allow an exploration of the hypothesis that relates infections (inflammatory alterations) during pregnancy, neurodevelopmental abnormalities, and an increased risk of neuropsychiatric diseases (21). Recent studies already have reported findings that suggest an immunological legacy imprinted on the neonate following maternal SARS-CoV-2 exposure, with potential far-reaching consequences (22–24).

A group of newborns of mothers infected by SARS-CoV-2 will present biological markers suggestive of presenting an abnormal inflammatory state (inflammation signature) that puts them at risk of suffering neurodevelopmental alterations. Inflammatory alterations in newborns will be related to clinical variables such as the time (gestational age) of maternal infection and its severity and, based on previous literature, alterations in the innate immune response will be associated with the clinical phenotype of the newborn.

## Research hypothesis

All this information, with different degrees of evidence, leads us to take advantage of the exceptional situation produced by the COVID-19 pandemic, to try to answer the following questions: (i) To understand the inflammatory / immune status of the newborn (NB) of mothers infected by SARS-CoV-2?; (ii) is there a relationship between the clinical characteristics of the maternal infection (severity / time of infection / etc…) and the inflammatory status of the newborn?; (iii) could these features increase the vulnerability to develop CNS alterations at an early age, neurodevelopmental alterations during the first years of life and eventually during adult life?; (iv) How is the SARS-CoV-2 infected mother's placenta altered? Do the placental alterations COVID-19 mediated contribute to developing CNS alterations? (v) is the infection associated with phenotypes obtained from the neurological and neurodevelopmental clinical evaluation (hypotonia, clumsiness, impaired communication and sociability) in newborns, and at 6 and 12 months and 24 months?

## Methods and analysis

### Study design and data sources

A prospective study of cohorts of SARS-CoV-2 infected and uninfected pregnant women during the gestational period and of a cohort of newborns of these pregnancies, with a prospective follow-up of 24 months. Confirming to international standards for research ethics, this project was approved by the local institutional review board (the Clinical Research Ethics Committee of Hospital Virgen del Rocío). Patients meeting inclusion criteria provided written informed consent prior to their inclusion in the study.

### Setting and procedures

The study will be carried out at the Hospital Universitario Virgen del Rocío (HUVR) and the Instituto de Biomedicina de Sevilla (IBiS). The multidisciplinary research team will include various specialists in pediatrics, psychiatry, gynaecology, clinical psychology, primary care, as well as scientists who are experts in basic and translational research, immunology and infectious diseases. The SARS Cov-2 uninfected women will be taken in primary care centres.

HUVR has a protocol for care for pregnant women in cases of COVID-19. All SARS-CoV-2 infected women attend Gynaecology consultations, specifically created (pregnancy and COVID-19 +). Once a case has been identified, a patient will be invited to participate, after confirming inclusion and exclusion criteria and signing the informed consent. In parallel, a SARS Cov-2 uninfected women with the same gestation time will be included. The same tests will be performed, plus a test that confirms non-infection by SARS-CoV-2 or previous COVID-19 infection. All ultrasound tests will be performed at the HUVR for homogenization. All pregnant women who acquire the COVID-19 and give birth during the inclusion period (1 of January of 2021 to 31 of august of 2022) and who are treated at the HUVR gynaecology and obstetrics service [pregnant (+) COVID-19] will be included. In addition, the mother / baby dyad will be followed up for 24 months postpartum.

**Figure 1:**
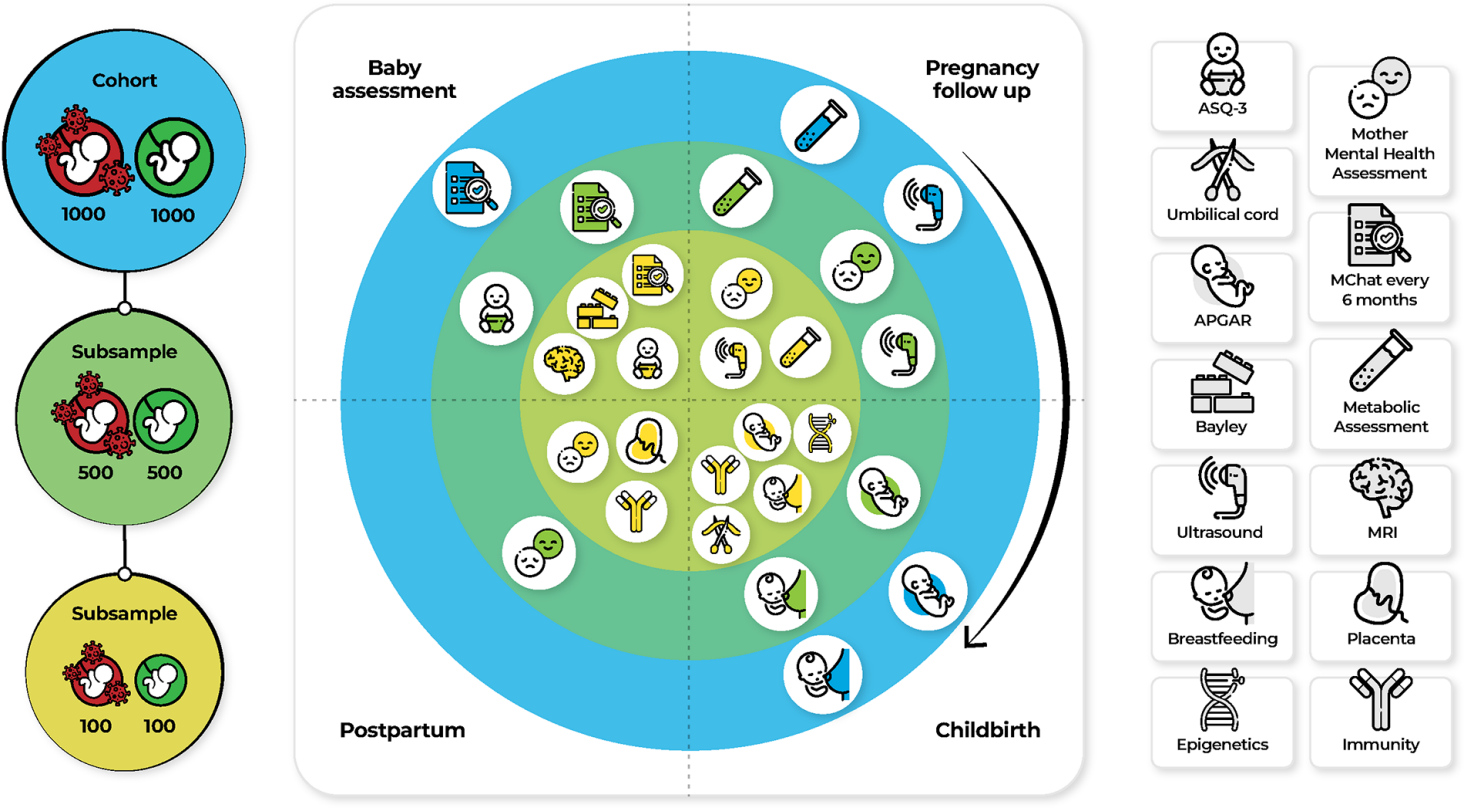
Assessment and measures for the specific aims in three samples. Abbreviations: APGAR, Appearance, Pulse, Grimace, Activity, and Respiration; ASQ-3, Ages and stages questionnaires; M-CHAT, Modified Checklist for Autism in Toddlers, Revised, with Follow-Up; MRI, Magnetic Resonance Imaging.

The cohort was divided in two groups: 1,000 SARS-CoV-2 infected women and 1,000 SARS Cov-2 uninfected women. (Figure 1, left panel). Two subsample (Subsample A and B) (Figure 1, left panel) were chosen to adhere to our interest in the relationship between prenatal stress and anxiety, proinflammatory status during pregnancy and neurodevelopmental disorders (Figure 1, middle panel); All women from the cohort will be assessed, women and children from subsample A will be assessed with mental health questionnaires, using the M-CHAT and Bayley assessment, and subsample B will be additionally assessed biologically. All these information will be presented as working packages (WP) (see WP 3, 4, 6,) (Figure 1, middle and right panel).

### Study group

The SARS CoV-2 infected women cohort inclusion criteria are: (i) Pregnant and SARS CoV-2 infection; (ii) Over 18 years old; (iii) Ultrasound-confirmed pregnancy; (iv) Suffering COVID-19 (clinical criteria of a suspected case and with a positive PCR). (v) Asymptomatic person with a positive PCR with negative Immunoglobulin G (IgG)). The SARS CoV-2 infected women cohort exclusion criteria are: (i) Presents alcohol abuse during pregnancy; (ii) Other concomitant causes of risk of demonstrated neurodevelopmental disorders; (iii) Presents drug abuse except tobacco during pregnancy; (iv) under 18 years of age.

### Control group

The SARS CoV-2 uninfected women cohort inclusion criteria are: (i) Over 18 years old; (ii) Ultrasound-confirmed pregnancy. The SARS Cov-2 uninfected women cohort exclusion criteria are: (i) Presents alcohol abuse during pregnancy; (ii) Other concomitant causes of risk of demonstrated neurodevelopmental disorders; (iii) presents drug abuse except tobacco during pregnancy; (iv) under 18 years of age. If during the pregnancy follow-up, a SARS Cov-2 uninfected women presents a SARS-CoV-2 infection, it would be assigned to the group of SARS CoV-2 infected women.

### Sample size calculation

Sample size was calculated based on the reported data about the prevalence of neurodevelopmental disorders in children 58/100.000 (25). In order to obtain a difference of 3% between exposed and unexposed and according to the parameter choices for a desired power of 0.80% and 95% confidence level, we estimated that we would need 734 participants distributed over 367 SARS CoV-2 infected women and 367 SARS Cov-2 uninfected women for cohort A. Sample size analysis was conducted using Epidata software (26).

### Statistics

The Kolmogorov–Smirnov test will be used to examine the normality of the data and the Levene test will be used to analyse the homogeneity of variances. If the distribution of the variables does not follow a normal law, non-parametric tests will be used for its analysis (W-Wilcoxon Signed-Rank test for paired and dependent data, U-Mann Whitney test for independent data). The association between the Bayle III score and pro-inflammatory marker measures will be made through Pearson or Spearman correlation analysis, as appropriate. The dichotomised score comparison of the neonatologist's evaluation (normal vs. abnormal) between groups (infected vs. uninfected) will be carried out by applying the Pearson *χ*2 test. To compare the presence of pro-inflammatory markers between the two groups (infected vs. uninfected), a comparative t-student or Mann-Whitney test will be performed, as appropriate. The presence of pro-inflammatory markers in the group of newborns of mothers infected by SARS-CoV-2 will be examined with a two-tailed hypothesis at a power of 80% and an *α* level of 5%. All the analyses will be performed with version 25.0 SPSS Inc. To explore the hypothesis that the biological alterations of interest play a causal role in the phenotypic alterations, mediation analyses will be performed exploring whether the inflammatory and immunological alterations (mediators collected at birth) mediate the relationship between exposure (SARS-CoV-2-infected mothers) and abnormal scores on the Essence questionnaire, ASQ-3 questionnaire, M-CHAT and Bayley III scale at 6 and 12 months.

### Core variables and measures for the specific aims

The main variable of the proposal will be the clinical and immunological characterisation of the newborn of (+) COVID-19 mothers, with neurological, neurodevelopmental, anthropometric, and immunological evaluations at birth, at 6 months and at 12 months, stratified by the week of pregnancy where the mother was infected by SARS-CoV-2. Table 1 In order to create two pregnant women clinical cohorts, the project is split into seven work packages (WP):

**Table 1:** Measures and test administered to pregnant women and their newborns.

		Pregnancy	childbirth	6 months	12 months	>18 months
Mother	Mental health assesment	BSI, PSS, PDQ, STAI, EQ-5D	Breastfeeding advice	SEPD, PBQ BSI, Post Covid Neuropsychiatric Symptom Questionnaire Ad Hoc, ASSIST, Addressing social determinants of health, BSES-SF	Impact of Event Scale Revised,Parental Stress,EQ-5D STAI	
	Medical records	Current pregnancy Questionnaire, Health history questionnaire, Ultrasound (heart rate,biparietal diameter, abdominal circumference, transcerebral diameter, nuchal fold, femur length, fetal movements, fronto occipital diameter, posterior ventricle, cisterna magna, abdominal perimeter), blood pressure, heart rate.	Type of delivery Small for gestational age (percentile), intrauterine growth restriction, presence of morbidities, description of morbidities.			
	Biological samples	Metabolic assessment: hemogram, coagulation, tryglicerides, cholesterol, HDL, LDL, glucemya, creatinine, urea, glomerular rate IL-17A (CTLA-8), IL-18, IL-23, IL-6, IL-12p70, IL-9, TNF-α, IL-7, IL-10, IFN-*γ*, IFN-α, IL-1RA, IP-10 (CXCL10), IL-8 (CXCL8),and ENA-78 (CXCL5) soluble cytokine levels. Monocyte subsets, phenotype marker expression as CD40, CD11b, CD142, CX3CR1, CD49d, CCR2, CCR5, TLR2 and TRL4, and intracellular cytokines IL-6, IL-1α and TNF-α from monocytes previously stimulated *in vitro*. Dendritic cells subsets and phenotype marker expression as CD4, CD86, CD81, CCR7, Integrin-*β*7, PD-L1 and IDO. T-lymphocytes subsets, Tregs (CD127low FOXP3+) and phenotype marker expression as CD57, CD28, CCR5, CD38 and HLA-DR. IgG/IgM				
Baby	Neurodevelopment assessment		APGAR	ASQ-3 Essence	Bayley ASQ-3 Essence IBQ	M-chat MRI
	Medical records		Weight, height, head circumference, gestational age, fundus, otoacoustic emissions, neuromotor examination			NDD diagnosis ASD diagnosis
	Biological samples		Methilation EWAS IL-17A (CTLA-8), IL-18, IL-23, IL-6, IL-12p70, IL-9, TNF-α, IL-7, IL-10, IFN-*γ*, IFN-α, IL-1RA, IP-10 (CXCL10), IL-8 (CXCL8),and ENA-78 (CXCL5) soluble cytokine levels. Monocyte subsets, phenotype marker expression as CD40, CD11b, CD142, CX3CR1, CD49d, CCR2, CCR5, TLR2 and TRL4, and intracellular cytokines IL-6, IL-1α and TNF-α from monocytes previously stimulated *in vitro*. Dendritic cells subsets and phenotype marker expression as CD4, CD86, CD81, CCR7, Integrin-*β*7, PD-L1 and IDO. T-lymphocytes subsets, Tregs (CD127low FOXP3+) and phenotype marker expression as CD57, CD28, CCR5, CD38 and HLA-DR.			

BSI, Brief Symptom Inventory; PSS, Perceived Stress Scale; PDQ, Prenatal Distress Questionnaire spanish validated.

STAI, Quality of Life-5 Dimensions spanish versión, EQ-5D; SEPD, Edinburgh Postnatal Depression Scale; PBQ, Postpartum Attachment Questionnaire; ASSIST, Alcohol, Smoking and Substance Involvement Screening Test; BSES-SF, Breastfeeding Self-efficacy Scale-Short Form; IBQ, Infant Behavior Questionnaire; EWAS, Epigenomic wide assocaition study; NDD, Neurodevelopmental disorder; ASD, Autism Spectrum Disorder.

### WP1: Pregnancy, birth and foetus assessment

Baseline data will be collected in relation to physical health history, pregnancy, ultrasound (heart rate, biparietal diameter, abdominal circumference, transcerebral diameter, nuchal fold, femur length, foetal movements, fronto occipital diameter, posterior ventricle, cisterna magna, abdominal perimeter), and biological samples, and a follow-up will be carried out every 3 months, which will coincide with those proposed by the protocol of the gynaecology service. The following variables will be measured: blood type, obstetric history (pre-eclampsia, threatened abortion, gestational diabetes, threatened preterm birth, intrauterine growth retardation, chorioamnionitis, eclampsia); blood pressure, heart rate, cholesterol, triglycerides, glycaemia, drugs consumption, history of maternal inflammatory diseases and vaccination in pregnancy. Additionally, during labour, the following data will be collected: induced childbirth, spontaneous, instrument-assisted vaginal delivery, placenta, breastfeeding advice, early breastfeeding, and complications of childbirth.

### WP2: Mental health

Pregnant women and postnatal dyads (mothers and newborns) from cohort B will be evaluated through interviews conducted by the researcher. Scales and questionnaires will be applied with which the antecedents, social and psychological factors, and perceived stress during pregnancy will be exhaustively evaluated. Reactive psychosis cases have been reported as consequence of emotional stress related to SARS-CoV-2 infection (27). Brief Symptom Inventory (BSI) (28), State Trait Anxiety Inventory (STAI) (29), Perceived Stress Scale (PSS) (30), Prenatal Distress Questionnaire spanish validated (PDQ) (31), and Quality of Life-5 Dimensions spanish version (EQ-5D) (32) will be used. At 6 months, the following questionnaires will be performed: Edinburgh Postnatal Depression Scale (SEPD) (33), Postpartum Bonding Questionnaire (PBQ) (34), Brief Symptom Inventory (BSI) (28), Alcohol, Smoking and Substance Involvement Screening Test (ASSIST) (‘The Alcohol, Smoking and Substance Involvement Screening Test (ASSIST): (35) / Addressing social determinants of health (36). Neuropsychiatric Symptoms will be assessed with Post-Covid Neuropsychiatric Symptom Questionnaire after 6 months of infection (37, 38) (Table 2). Additionally, at 12 months, stress will be measured using psychological questionnaires such as: Parental Stress Scale (PSI-SF) (39), Impact of Event Scale-Revises (IES-R) (40).

**Table 2:** Post COVID neuropsychiatric symptom questionnaire.

Difficulty breathing or shortness of breath
Tiredness or fatigue
Symptoms that get worse after physical or mental activities (also known as post-exertional malaise)
Difficulty thinking or concentrating (sometimes referred to as “brain fog”)
Cough
Chest or stomach pain
Headache
Fast-beating or pounding heart (also known as heart palpitations)
Joint or muscle pain
Pins-and-needles feeling
Diarrhea
Sleep problems
Fever
Dizziness on standing (lightheadedness)
Rash
Mood changes
Change in smell or taste

### WP3: Inflammation

The main objective is to achieve: prognostic immunologic factors associated with cognitive impairment in children born of SARS-CoV-2-infected mothers. The integration of clinical and immunological data could elucidate immunological signatures influencing cognitive impairment in newborns of mothers infected with SARS-CoV-2. Cytokine quantification in plasma, a proinflammatory functional profile in monocytes, and a deep PBMC immunophenotyping (monocytes, dendritic cells and T-cells) using PBMCs from mothers with different COVID-19 symptomatology and their children will be evaluated. Firstly, IL-17A (CTLA-8), IL-18, IL-23, IL-6, IL-12p70, IL-9, TNF-α, IL-7, IL-10, IFN-*γ*, IFN *α*, IL-1RA, IP-10 (CXCL10), IL-8 (CXCL8), and ENA-78 (CXCL5) cytokines levels will be assayed in plasma samples utilising different kits according to the manufacturer's instructions.

Secondly, to proinflammatory functional profile in monocytes, PBMCs will be stimulated *in vitro* for 5 h at 37 °C with 0.5 *μ*l/ml of lipopolysaccharide (LPS, Invivogen) in R-10 medium, including other condition with PBMCs without any stimulation as a negative control. Monensin (Golgi Stop, BD Biosciences) was added to all experimental conditions. Intracellular cytokines IL-6, IL-1*α* and TNF-α will be analysed by flow cytometry. Finally, PBMC immunophenotyping will be assayed. For monocyte immunophenotyping, PBMCs will be evaluated for viability using LIVE/DEAD Fixable Violet Dead Cell Stain Kit. CD3, CD19, CD20, CD56, CD14, CD16 and HLA-DR antibodies will be used to assess lineage. CD40, CD11b, CD142, CX3CR1, CD49d, CCR2 and CCR5 antibodies will be used to assess activation and cell adhesion surface markers. Toll-like receptor (TLR)-2 and TLR4 antibodies will be used to assess TLR surface expression. For dendritic cells immunostaining, PBMCs will be evaluated for viability using LIVE/DEAD fixable Aqua Blue Dead Cell Stain. Lin-22 (CD3, CD19, CD20, CD14 and CD56), HLA-DR, CD11c, CD16, CD123, CD1c, CD4 and CD141 antibodies will be used to evaluate lineage. CD86 and CD81 antibodies will be used to assess activation. CCR7 and Integrin-*β*7 antibodies will be used to evaluate cellular homing and PD-L1 and IDO will be used to assess intracellular expression of suppression markers. T lymphocyte immunophenotyping will include staining with LIVE/DEAD fixable Aqua Blue Dead Cell Stain for viability and antibodies against CD14, CD19, CD56, CD8 and CD3 to evaluate lineage and CD27 and CD45RA to determine their subsets pending on maturation. CD57, CD28, CCR5, CD38 and HLA-DR antibodies will be used to assess senescence and activation, and survival and FOXP3 and CD127 to assess regulatory T cells (Tregs). Multiparametric flow cytometry was performed on an LRS Fortessa flow cytometer using FACS Diva software (BD Biosciences). Data were analysed using the FlowJo 10.7.1 software (Treestar, Ashland, OR).

### WP4: Placenta

The placenta is considered the main modulator of maternal-foetal interchange and plays a key role in foetal development (18). Furthermore, the placenta has intrinsic functions. For example, the serotonin synthesised by the placenta is necessary for foetal neurodevelopment, including processes such as cortical neurogenesis, migration and initial axon targeting (41). Disruption of the placental-mediated serotoninergic interchange may occur in prenatal maternal infection and/or stress causing impaired development and, thereby, contributing to neurodevelopmental disorders such as ASD and schizophrenia. Recently (42), have evidenced that placentas from women with SARS-CoV-2 display alterations in their immune collection, affecting the neonatal immune system. Their results indicate that SARS-CoV-2 infection differentially impacts the transcriptome of the mother and the neonate and that such changes are partly shared with those in the placental tissues. How these placental alterations impact in the foetal development and if the serotoninergic interchange is affected remain to be clarified and require further investigations. Accordingly, our research group is currently collecting placental samples, from control and SARS-CoV-2 infected pregnant women, in order to establish a biobank for our research goals. To achieve an optimal sampling, we are following the recommendations suggested in the collection protocol of placental samples from (43).

### WP5: Neonatal assessment and neurodevelopment

The following data will be collected from medical records: sex (0, female; 1, male), gestational age (weeks), birth weight (grams), Apgar at minute 1 and 5 (range: 1–10), head circumference (centimetres), length (cm), type of delivery (0, spontaneous vaginal; 1, operative vaginal; 2, elective caesarean; 3, emergency caesarean), small for gestational age (percentile), intrauterine growth restriction (0, no; 1, yes), presence of morbidities (0, no; 1, yes), description of morbidities. The specific follow-up protocol will be similar in the most relevant aspects to the one used for the NB less than 1.500 grams (g) or less than 32 weeks of gestation age (Pallás Alonso et al., 2018) which includes: weight, height, head circumference, gestational age, fundus, otoacoustic emissions, and neuromotor examination. COVID-19 diagnosis will be performed by PCR / NB serum. At 6 months the following questionnaires will be performed by the parents: Essence Q Rev (44)./ Ages and stages questionnaires (ASQ-3) (45). At 12 months, ASQ-3 and the Bayley III scale (46) will be performed. Additionally we will collect data about breastfeeding duration (6 months and 12 months) and Breastfeeding Self-efficacy Scale-Short Form (BSES-SF) (47). At 18 months, Modified Checklist for Autism in Toddlers, Revised, with Follow-Up (M-CHAT) (48) questionnaires will be performed and ASD assessment by a specialist team when m-chat is positive (Table 1).

### WP6: Infectiology

Identification of SARS-CoV-2 RNA will be performed in plasma samples of mothers (peripheral blood) and babies (umbilical cord blood) and, in the positive cases, the viral load will be determined. Identification of SARS-CoV-2 will be carried out in placenta samples, as well as the viral load. SARS-CoV-2 viability in blood from the mothers and babies and in placenta will be analysed in cellular cultures. In addition, the quantification of IgG/IgM antibodies will be evaluated in the serum samples of the patients (mothers and babies).

Placenta tissue samples will be assayed by electron microscopy to visualise the intracellular location of SARS-CoV-2 virions. In addition, immunohistochemical staining will be performed on formalin-fixed and paraffin-embedded tissue sections to evaluate the expression and distribution of the SARS-CoV-2 antigens (49). We will measure severity signs of COVID-19 during pregnancy, according to the WHO clinical progression scale (Table 3) (50).

**Table 3:** WHO clinical progression scale.

Patient State	Descriptor	Score
Uninfected	Uninfected; no viral RNA detected	0
Ambulatory mild disease	Asymptomatic; viral RNA detected	1
	Symptomatic; independent	2
	Symptomatic; assistance needed	3
Hospitalized: moderate disease	Hospitalized; no oxygen therapy^a^	4
	Hospitalized; oxygen by mask or nasal prongs	5
Hospitalized: severe diseases	Hospitalized; oxygen by NIV or high flow	6
	Intubation and mechanical ventilation, pO2/FiO2 _150 or SpO2/FiO2 _200	7
	Mechanical ventilation pO2/FIO2 < 150 (SpO2/FiO2 < 200) or vasopressors	8
	Mechanical ventilation pO2/FiO2 < 150 and vasopressors, dialysis, or ECMO	9
Dead	Dead	10

ECMO, extracorporeal membrane oxygenation; FiO2, fraction of inspired oxygen; NIV, non-invasive ventilation; pO2, partial pressure of oxygen; SpO2, oxygen saturation.

^a^ If hospitalized for isolation only, record status as for ambulatory patient.

### WP7: Neuroimaging

Twenty 3- years old children from infected mothers group will be scanned with Magnetic Resonance Imaging (MRI) for establishing brain structural alterations. During a single session, two different sequences will be acquired: (i) Diffusion Tensor Image, which will allow establishing inflammatory and microstructural alterations of the white matter in areas associated with ASD (such as limbic-cortical pathway) and (ii) MPRAGE T1, standard sequence deriving morphometric measurements such as volume, cortical thickness and gyrification which has been also associated with ASD. MRI will be processed using established and validate pipelines implemented in Free surfer (https://surfer.nmr.mgh.harvard.edu/). As there will be no control group, we will use a strategy for data normalization that will exploit images of individuals in the same age group and gender collected in public data using the methodology described in Bethlehem et al., 2022 (51). Thanks to its online tool (https://brainchart.shinyapps.io/brainchart/) it will be possible effectively detect abnormal deviations of brain maturational trajectories of our cohort of 3-years old children.

### WP8: Epigenetics

The genome methylation pattern of newborns will be determined from blood samples collected at the time of delivery. The samples will be outsourced and processed. To detect the methylation pattern, the Illumina Infinium Human MethylationEPIC BeadChip kit high-throughput array will be used. From the data obtained, differential methylation analysis will be performed with GenomeStudio and R software.

## Results

This is a protocol report and no analyses have been conducted yet, being currently at, our study is in the recruitment process step. At the time of this publication, we have identified 1,060 SARS-CoV-2 infected mothers and all have already given birth. From the total of identified mothers, we have recruited 537 SARS-COV-2 infected women and all of them have completed the mental health assessment during pregnancy. We have collected biological samples from 119 mothers and babies. Only 214 babies are 12 months old, 385 babies are between 6 and 11 months old and 431 babies are younger than 6 months. Additionally, we have recruited 390 non-infected pregnant women.

## Discussion

In this study, we will create two epidemiological cohorts (clinical and biological information) of pregnant women in the health area of the HUVR: a cohort of pregnant women (+) COVID-19 and a cohort of pregnant women (-) COVID-19 in which we will investigate the signature status of inflammation during pregnancy and in their offspring. In addition, we will evaluate: (i) the risk of developing psychosis or postpartum depression in mothers (+) COVID-19, we will identify signs and symptoms of neurodevelopmental disorders in neonates born to mothers (+) COVID, both at birth and at 6 and 12 months of life; (ii) we will analyse the relationship between the inflammatory signature (elevated inflammatory state) and developmental disorders or the appearance of neuropsychiatric disorders during the first years of life; (iii) we will obtain umbilical cord blood cells with stem cell phenotype with the potential to differentiate into neural lineages. (iv) we will establish a systematic follow-up protocol for the HUVR cohort of COVID-19 newborns as children at a high risk of developing neurodevelopmental disorders.

### Expected results

Neurodevelopmental alterations will be evidenced by clinical signs in the first year of life: (i) difficulty directing their gaze in the direction in which another person is looking or pointing; (ii) a lack of joint attention: gaze does not alternate between an object and the adult showing or holding it; (iii) absence of communicative gestures; (iv) absence of socio-communicative babbling; (v) absence of social smile and spontaneous imitation; (vi) lack of interest in playing; (vii) lack of response when called by name; (viii) abnormal muscle tone, posture and movement patterns. H1. Analysis of biological samples from mothers and newborns will show changes in cytokine balance in T-helper Type 1 (TH1) cells (TNF-a, IFN-g) and TH2 cytokines (interleukins IL-4, IL-5, IL-6). More significant changes in the phenotype (monocyte function and activation of the umbilical cord sample) will be observed in the cases of pregnant women who were infected by SARS-CoV-2 in the first trimester of pregnancy. H2. Babies of mothers who were infected by SARS-CoV-2 will present high scores in the neurological evaluation (evaluating motor, language and motor function performed with the Bayley III scale) at 12 months. Regardless of the trimester in which they had the infection, they will present a greater alteration in neurodevelopment measured according to this scale and children will be considered at higher risk of developing a neuropsychiatric disorder in adulthood.

It has been reported that first and second trimester maternal SARS-CoV-2 infection is a risk factor for preterm birth and stillbirth (52, 53), and that birth during the pandemic, but not being infected by SARS-CoV-2, is associated with alterations in neurodevelopment at age 6 months (54). SARS-CoV-2 during late pregnancy did not increase the risk of developmental delay of the offspring 3 months after delivery. However, SARS-CoV-2 may have indirect effects on early childhood development by increasing mother-infant separation. The current worldwide research is to focus on exploring the role of target gene methylation levels in mediating the association between maternal prenatal stress related to the COVID-19 emergency and infant developmental outcomes (55) and generating knowledge about the psychological consequences of pandemics on pregnant individuals and to point toward prevention and intervention targets (56).

In utero exposure to infections have been shown to increase the risk of developing ASD or other neuropsychiatric diseases (1). Our proposal seeks to complete an innovative collaboration strategy between clinical and basic researchers that look toward interdisciplinary and integrative research to answer a research question that transcends the borders of the areas of knowledge involved individually and which is of high social importance. It is too soon to observe the consequences of *in utero* SARS-CoV-2 exposure on neurodevelopment; however, it is important to follow children exposed to SARS-CoV-2 *in utero* to determine the risk of the long-term neurodevelopmental outcomes.

In order to homogenise the procedures, we are publishing the protocol of the *SIGNATURE* project that started in January 2021. The main objective of publishing the protocol is to discuss with other research groups the methods, and sharing similar instruments of measure in order to compare results and obtain a large sample around the world of pregnant women infected by SARS-CoV-2 and their newborns.
